# The Effect of Older Adults’ Volunteering on Relational Satisfaction

**DOI:** 10.1093/geroni/igaa057.1314

**Published:** 2020-12-16

**Authors:** Meeryoung Kim

**Affiliations:** Daegu University, Gyeongsan, Republic of Korea

## Abstract

Longevity is increasing in what is called the centenarian society. However, the average retirement age of Korea is the lowest among OECD countries. Because of increasing longevity, older adults need activities after retirement. Volunteering can be a substitute that allows Korean older adults to find a social identity. This study examined older adults’ volunteering and how many kinds of volunteering affected relational satisfaction differently. This study used the 6th additional wave of the Korean Retirement and Income Study (2016). The target population of this study was ages over 60 and the sample size was 280. For data analysis, multiple regressions were used. Demographic variables were controlled. As for independent variables, reasons for volunteering whether they were motivated for self or for others were used. For dependent variables, relational satisfaction, such as family, human relation and overall life satisfaction was used. Volunteers’ health is an important factor for relational satisfaction. If volunteering was self-motivated, satisfaction of both family and human relations were negatively affected. Reason for others also affected satisfaction of family and human relations negatively. Volunteering initiated by others increased satisfaction of family and human relations. Doing more than one kind of volunteering affected both satisfaction of family and human relations. For overall life satisfaction, the effect of volunteering for oneself was lower than other reasons. These findings implied that reasons for volunteering affected relational satisfaction differently. In addition, the activities of volunteering, such as taking part in one or more had different effects.

